# Leisure-related sedentary time and physical activity with myocardial infarction incidence: a prospective cohort study

**DOI:** 10.3389/fnut.2026.1902990

**Published:** 2026-09-09

**Authors:** Chang-Qing Sun, Meng-Ting Liu, Qian-Yu Zhou, Rong Su, Xiao-Fang Dong, Jia-Jun Chen, Qiang Zhang

**Affiliations:** 1 School of Nursing and Health, Zhengzhou University, Zhengzhou, China; 2 College of Public Health, Zhengzhou University, Zhengzhou, China; 3 Department of Endocrine and Metabolic Medicine, The First Affiliated Hospital of Zhengzhou University, Zhengzhou, China; 4 Department of Human Resources, The First Affiliated Hospital of Zhengzhou University, Zhengzhou, China

**Keywords:** incidence, ISM, leisure-related sedentary time, myocardial infarction, physical activity

## Abstract

**Objectives:**

This study aimed to comprehensively investigate the associations between leisure-related sedentary time, physical activity (PA), and the incidence of myocardial infarction (MI) and its subtypes.

**Methods:**

This cohort study included 475,874 individuals free of MI at baseline. Daily behavioral data—including leisure-related sedentary time, PA, and sleep duration—along with baseline demographic characteristics were collected. Three multivariable-adjusted Cox proportional hazards models were constructed to assess the association between leisure-related sedentary time and incident MI. Additionally, a mutually adjusted Cox model incorporating all four behaviors simultaneously was used to estimate hazard ratios for inter-behavior comparisons via coefficient contrasts, evaluating whether higher PA or sleep durations, independent of other measured behaviors, were associated with reduced MI risk.

**Results:**

Compared with the reference group, individuals with leisure-related sedentary time exceeding 4 h per day had an increased risk of MI and its subtypes after full multivariable adjustment. Mutually adjusted analyses demonstrated inverse associations with MI risk across all behaviors. Coefficient contrasts indicated that a 60-min higher duration of sleep, LPA, or MVPA—holding other measured behaviors constant—corresponded to hazard ratios of 0.967 (95% CI: 0.954–0.980) for sleep, 0.987 (95% CI: 0.964–1.011) for LPA, and 0.974 (95% CI: 0.953–0.995) for MVPA. Consistent patterns were observed for 30-min and 15-min contrasts, with estimated risk reductions ranging from approximately 1–6% across all comparisons.

**Conclusion:**

Prolonged leisure-related sedentary time is associated with an increased risk of MI, STEMI, and NSTEMI. Independent of other daily behaviors, higher durations of PA and sleep are inversely associated with MI incidence, with benefits evident even at modest (15–30 min) differences in daily duration.

## 1 Introduction

Coronary heart disease (CHD) is a leading cause of cardiovascular disease (CVD) morbidity and premature mortality worldwide (1). Myocardial infarction (MI), the most common and serious manifestation of CHD, affects approximately 0.5 million individuals annually as a first event and 0.2 million as recurrent acute episodes (2). Notably, the global burden of CVD and acute MI has shifted substantially toward low- and middle-income countries, which now account for approximately 80% of CVD-related deaths globally (1, 3). Based on electrocardiographic findings, MI is further classified into ST-elevation myocardial infarction (STEMI) and non-ST-elevation myocardial infarction (NSTEMI), each with distinct clinical implications.

Daily time use—comprising leisure-related sedentary behavior, physical activity (PA), and sleep—represents a modifiable set of health determinants. Current guidelines from the World Health Organization (WHO) (4) and the United States (5) recommend that adults accumulate at least 150 min/week of moderate-to-vigorous PA (MVPA), limit sedentary time to fewer than 8 h/day, and obtain 7–9 h/day of good-quality sleep for optimal health. However, pooled analyses of data from approximately 1.9 million adults and 1.6 million adolescents indicate that 28% of adults (6) and 81% of adolescents (7) fail to meet WHO PA recommendations.

Surveillance data further reveal high population-level sedentary exposure: approximately 30% of adults in the UK report more than 6 h/day of sedentary time on weekdays, rising to 37% on weekends. Similarly, National Health and Nutrition Examination Survey (NHANES) 2015–2016 data showed that 25.7% of US adults reported total sedentary time exceeding 8 h/day (8). Trend analyses of 51,896 US participants across NHANES cycles (2001–2016) documented stable television-viewing time among adolescents and young adults but significant increases in computer-based and total sedentary time across both age groups (9).

Extensive evidence links prolonged sedentary time to a range of non-communicable diseases (NCDs), including CVD (10, 11), type 2 diabetes (12), cancer (13), depression (14), and all-cause mortality (10), supporting the notion that reducing sedentary behavior confers multi-system health benefits. Longitudinal analyses from the UK Biobank further demonstrate joint associations of PA and sleep with all-cause mortality, wherein lower PA amplifies the adverse effects of poor sleep (15). Conversely, increasing MVPA to recommended levels has been shown to reduce risks of all-cause mortality, CVD mortality, and atherosclerotic CVD (16). These findings suggest that the detrimental association between leisure-related sedentary time and MI may be mitigated by higher PA levels.

Recent studies have applied isotemporal substitution models (ISMs) to quantify the health impact of reallocating sedentary time to PA of varying intensities across diverse outcomes (17–22). However, no prior investigation has specifically examined MI or its subtypes using this approach. To address this gap, the present study investigated the association between leisure-related sedentary time and incident MI, STEMI, and NSTEMI, and evaluated whether this association is modified when sedentary time is reallocated to PA of different intensities or to sleep.

## 2 Materials and methods

### 2.1 Study population and the outcomes

**Figure 1:**
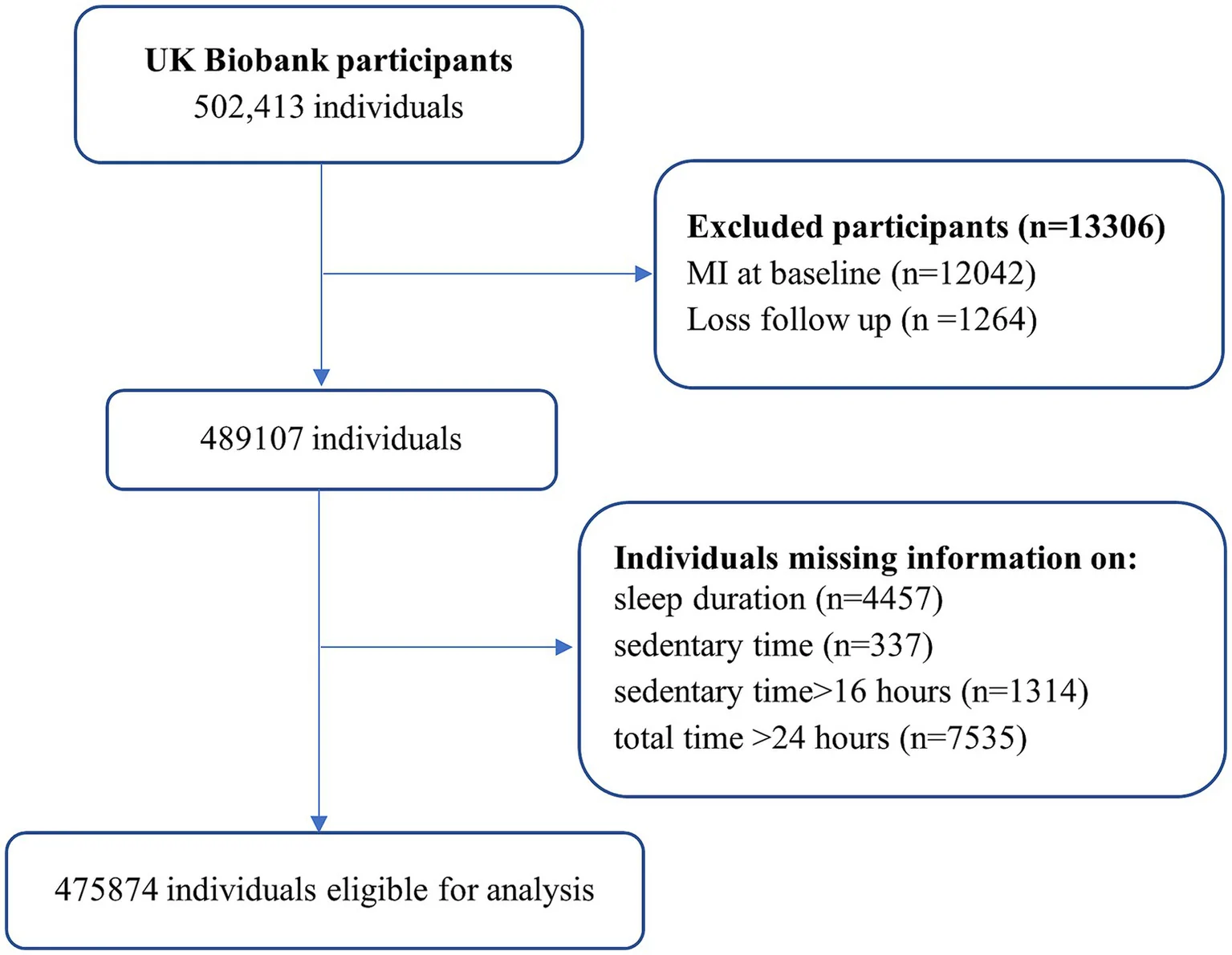
Flowchart of the participant selection.

The UKB dataset is a large prospective cohort study which consists of more than 502,413 individuals aged 40–69 years, with baseline assessments between 2006 and 2010 (23). The participants were recruited from 22 different assessment centers across England, Scotland and Wales in the UK; through a touch-screen questionnaire, participants provided their basic population characteristics, biological samples, genomics data and multiple health-related outcomes. In the current study, participants with MI at baseline (occurrence date was before the date of first attendance at a UKB assessment center, *n* = 12,042), those without follow-up information (*n* = 1,264), participants with missing information on sleep duration (*n* = 4,047) and sedentary time (*n* = 337) were excluded. In addition, we also excluded participants with sedentary time >16 h (*n* = 1,314) and total time >24 h (*n* = 7,535). Finally, a total of 475,874 individuals were included for the analysis (Figure 1).

This study included three outcomes: MI, STEMI, and NSTEMI. The primary outcome for this study is MI incidence, which was defined using UK Biobank algorithmically defined outcomes, derived from hospital admission records, death registry records, and excluding self-reported prevalent MI at baseline (Field 42,000). Subtypes were classified as STEMI (Fields 42,002) and NSTEMI (Fields 42,004); unspecified MI was retained in the primary outcome but excluded from subtype analyses. Follow-up time was started from the date of first attendance at the assessment center to the earliest date of MI, STEMI, and NSTEMI diagnosis, death, or the censor date based on the latest linkage cutoff date provided by UK Biobank at the time of data access (2022-2-23), whichever occurred first. This study was performed in compliance with the observational cohort guideline STROBE checklist.

### 2.2 Assessment of leisure-related sedentary time, PA and sleep duration

Leisure-related sedentary time, PA, and sleep were all collected using self-reported questions. Leisure-related sedentary time was assessed using three data fields: self-reported time spent watching television (TV), using a computer, and driving. Participants were asked “In a typical DAY, how many hours do you spend watching TV, using the computer and driving in leisure time”, and the sum of those three activities were defined as total sedentary time, then sedentary time was categorized into five groups as “≤2 h”, “2.1–4 h”, “4.1–6 h”, “6.1–8 h”, and “>8 h.”

Physical activity was measured using the validated International PA Questionnaire (IPAQ), participants were asked about the duration and frequency of three different intensity of activities: walk (frequency, Field ID: 864; duration, Field ID: 874), moderate activity (frequency, Field ID: 884; duration, Field ID: 894) and vigorous activity (frequency, Field ID: 904; duration, Field ID: 914). For each domain, weekly frequency (days/week) was multiplied by typical session duration (minutes). Total weekly minutes across all three domains were summed, then divided by 7 and 60 to derive average daily hours. Sleep time was assessed by asking “About how many hours sleep do you get in every 24 h? (Please include naps).”

### 2.3 Covariates

This study included three groups of covariates selected *a priori* based on a prespecified causal framework (Supplementary Figure S1). The first group included basic demographic information: age, gender, ethnic background, and Townsend deprivation index. The second group further included lifestyle and socioeconomic confounders: employment status (employed, unemployed, and retired), average total household income (less than 18,000, 18,000 to 30,999, 31,000 to 51,999, 52,000 to 100,000, and greater than 100,000), smoking and alcohol drinking status (never, previous, and current), and total physical activity time. The third group further included potential mediators of the behavior–MI pathway: use of cardiometabolic medications, BMI (kg/m^2^), diabetes (yes/no), cancer (yes/no), high blood pressure (yes/no), and depression (yes/no).

### 2.4 Statistical analyses

The baseline characteristics of the participants were described using mean and standard deviation (SD) for continuous data, and categorical data were reported as frequencies and percentages. Associations between different groups of leisure-related sedentary time and the outcomes were estimated using Cox proportional hazards models and 95% CIs; participants who reported sitting time ≤2 h were used as the reference group. Schoenfeld residuals method was applied to test the proportional hazards assumption; for variables that might have violated the PH assumption, a product term of time with that variable would be included in the model. Model 1 was adjusted for age, gender, ethnicity, and Townsend deprivation index. Model 2 was additionally adjusted for average total household income, employment status, smoking and alcohol drinking status, and total PA time. Model 3 was further adjusted for BMI, diabetes, cancer, high blood pressure, depression, and use of cardiometabolic medications.

Mutually adjusted behavioral associations. To evaluate whether the association between one behavior and MI risk was independent of other daily behaviors, all four continuously measured behavioral variables—sleep duration (hours/day), leisure sedentary time (sum of television viewing, computer use, and driving; hours/day), light physical activity (LPA; hours/day), and moderate-to-vigorous physical activity (MVPA; hours/day)—were entered simultaneously into a single Cox model. This mutually adjusted framework allows estimation of the hazard ratio for a given behavior while holding the other three measured behaviors constant at their observed values. The model was specified as:

$$h_{i}\;(t)=h_{0}\;(t)\exp(\beta_{1}\;X_{1i}+\beta_{2}\;X_{2i}+\beta_{3}\;X_{3i}+\beta_{4}\;X_{4i}+\theta ⊤Z_{i})$$

where $X_{1i}$ , $X_{2i}\;$ , $X_{3i},X_{4i}$ denote daily durations (in hours) of sleep, leisure sedentary time, LPA, and MVPA, respectively; $Z_{i}$ ​ represents the vector of Model 3 covariates excluding total PA time; and θ denotes the corresponding regression coefficients. All time variables were measured in hours per day. No total-time term was included because the measured behaviors do not constitute a complete 24-h composition.

Distribution of measured behaviors and residual time. The sum of the four continuously measured daily behaviors—sleep, leisure sedentary time, LPA, and MVPA—was calculated for each participant. The mean (SD), median, interquartile range, and range of this sum were reported. Residual time was defined as 1,440 min/day minus the sum, representing unmeasured waking activities (e.g., occupational sitting, socializing, and personal care). This residual was not explicitly modeled. Participants with a measured sum exceeding 1,440 min/day were excluded from the mutually adjusted analysis.

Coefficient contrasts for inter-behavior comparisons. To quantify the relative difference in MI risk between two behaviors, we computed hazard ratios as the exponential of the negative product of the time difference and the contrast of their regression coefficients:

$$HR_{A\to B}\;(\Delta )=\exp[-\Delta \times (\beta_{A}-\beta_{B})]$$

where $\beta_{A}$ and $\beta_{B}$ ​ are the natural-log hazard ratios per hour/day from the mutually adjusted model, and Δ is the reallocation magnitude (15 min, 30 min, or 1 h/day). Standard errors and 95% confidence intervals were derived from the variance–covariance matrix of ( $\beta_{A}$ ， $\beta_{B}$ ) using the delta method. These estimates represent hypothetical contrasts between behaviors rather than true within-person time exchanges, because the measured behaviors do not constitute a complete 24-h composition.

The association between different groups of leisure-related sedentary time and STEMI/NSTEMI was also assessed using the final model described above. To account for potential non-linearity between continuous variables and MI incidence, we assessed the dose–response associations of age, BMI, sedentary time, and sleep duration with MI (STEMI/NSTEMI) incidence outcomes using a restricted cubic spline model with five knots. Additionally, sensitivity analysis was conducted to test the robustness of the main results. First, to exclude the interference of reverse causality, we repeated the main MI incidence outcome models after excluding participants who had MI within the first 2 years of follow-up. To account for the competing risk of mortality, we used the Fine and Gray subdistribution model to explore the association between leisure-related sedentary time and outcome incidence. All analyses were performed using R version 4.3 (R Foundation for Statistical Computing). Cox models were fitted using the coxph function from the survival package. Competing-risk models used the cmprsk package. Restricted cubic splines were generated using the rcs function from the rms package. Variance–covariance matrices for coefficient contrasts were extracted using base R functions.

## 3 Results

### 3.1 Population characteristics

The participant selection procedure is illustrated in Figure 1. Table 1 shows the baseline characteristics of the study participants according to different groups of leisure-related sedentary time. Among the 475,874 participants included in this study, we documented 15,262 MI cases, 3,872 STEMI cases, and 7,913 NSTEMI cases. The median follow-up period was 13.1 years (IQR: 12.4–13.8) for MI incidence. Approximately 20% of the participants (92, 670) reported leisure-related sedentary time of more than 6 h per day. Compared to those with sitting time ≤2 h, those with longer sedentary times are more likely to be older, male, employed, current drinkers, and to have a higher Townsend deprivation index and BMI. Participants who spent longer time in leisure-related sedentary time are more likely to have lower income and a higher prevalence of diabetes, depression, cancer, and high blood pressure. For groups that spent 6.1–8 h and >8 h per day, the outcome incidence was reported to be higher than in the group with ≤2 h (Table 1). At baseline, the mean sum of the four measured behaviors was 13.8 h/day (SD = 3.1), with a median of 13.4 h/day (IQR: 11.7–15.6). The mean residual time was 10.2 h/day, accounting for approximately 42% of the day.

**Table 1:** Baseline characteristics of study patients by different leisure-related sedentary time.

Characteristic	Overall, *N* = 475,874*^1^*	≤2 h, *N* = 56,887*^1^*	2.1–4.0 h, *N* = 173,987*^1^*	4.1–6.0 h, *N* = 152,330*^1^*	6.1–8.0 h, *N* = 59,521*^1^*	>8 h, *N* = 33,149*^1^*
Age	56.44 (8.09)	55.41 (8.18)	56.04 (8.12)	57.20 (7.97)	57.28 (7.95)	55.28 (8.07)
Female	264,702 (56%)	37,839 (67%)	107,861 (62%)	81,052 (53%)	26,429 (44%)	11,521 (35%)
BMI	27.36 (4.76)	25.77 (4.47)	26.69 (4.46)	27.76 (4.66)	28.70 (4.95)	29.34 (5.33)
White	432,678 (91%)	49,032 (86%)	158,469 (91%)	140,853 (92%)	54,643 (92%)	29,681 (90%)
Household income						
Less than 18,000	106,983 (22%)	12,940 (23%)	34,386 (20%)	35,297 (23%)	15,662 (26%)	8,698 (26%)
18,000 to 30,999	120,638 (25%)	13,091 (23%)	42,879 (25%)	40,173 (26%)	15,983 (27%)	8,512 (26%)
31,000 to 51,999	124,277 (26%)	13,921 (24%)	46,538 (27%)	40,280 (26%)	15,177 (25%)	8,361 (25%)
52,000 to 100,000	97,882 (21%)	12,304 (22%)	39,352 (23%)	29,675 (19%)	10,407 (17%)	6,144 (19%)
Greater than 100,000	26,094 (5.5%)	4,631 (8.1%)	10,832 (6.2%)	6,905 (4.5%)	2,292 (3.9%)	1,434 (4.3%)
Employment status						
Unemployed	7,681 (1.6%)	834 (1.5%)	1,972 (1.1%)	2,394 (1.6%)	1,418 (2.4%)	1,063 (3.2%)
Employed	274,980 (58%)	36,139 (64%)	107,117 (62%)	81,013 (54%)	29,935 (51%)	20,776 (63%)
Retired	157,518 (33%)	14,636 (26%)	53,341 (31%)	58,283 (39%)	23,182 (39%)	8,076 (25%)
Others	31,323 (6.6%)	4,577 (8.1%)	10,088 (5.8%)	9,299 (6.2%)	4,463 (7.6%)	2,896 (8.8%)
Unknown	4,372	701	1,469	1,341	523	338
Smoking status						
Never	264,001 (55%)	35,119 (62%)	102,095 (59%)	81,804 (54%)	29,324 (49%)	15,659 (47%)
Previous	162,954 (34%)	16,370 (29%)	56,297 (32%)	55,035 (36%)	22,887 (38%)	12,365 (37%)
Current	48,919 (10%)	5,398 (9.5%)	15,595 (9.0%)	15,491 (10%)	7,310 (12%)	5,125 (15%)
Drinking status						
Never	20,819 (4.4%)	4,051 (7.1%)	7,378 (4.2%)	5,545 (3.6%)	2,336 (3.9%)	1,509 (4.6%)
Previous	16,553 (3.5%)	2,304 (4.1%)	5,452 (3.1%)	4,925 (3.2%)	2,325 (3.9%)	1,547 (4.7%)
Current	438,502 (92%)	50,532 (89%)	161,157 (93%)	141,860 (93%)	54,860 (92%)	30,093 (91%)
Townsend deprivation index	−1.35 (3.06)	−0.76 (3.29)	−1.50 (2.97)	−1.56 (2.95)	−1.25 (3.11)	−0.76 (3.29)
Depression	112,010 (24%)	13,433 (24%)	38,886 (22%)	34,914 (23%)	15,196 (26%)	9,581 (29%)
Cancer	109,729 (23%)	11,858 (21%)	39,086 (22%)	36,947 (24%)	14,728 (25%)	7,110 (21%)
High blood pressure	112,435 (24%)	10,338 (18%)	36,846 (21%)	38,515 (25%)	16,969 (29%)	9,767 (29%)
Diabetes: *n* (%)	23,198 (4.9%)	2,070 (3.6%)	6,289 (3.6%)	7,756 (5.1%)	4,186 (7.0%)	2,897 (8.7%)
Sleep duration	7.15 (1.09)	7.15 (1.12)	7.16 (1.03)	7.17 (1.09)	7.15 (1.17)	7.04 (1.21)
LPA	0.93 (1.18)	1.02 (1.31)	0.98 (1.24)	0.93 (1.17)	0.85 (1.04)	0.67 (0.80)
MPA	0.75 (1.04)	0.82 (1.16)	0.79 (1.09)	0.75 (1.04)	0.68 (0.92)	0.53 (0.70)
VPA	0.29 (0.42)	0.32 (0.47)	0.30 (0.43)	0.29 (0.42)	0.27 (0.39)	0.25 (0.35)
MI	15,262 (3.2%)	1,328 (2.3%)	4,581 (2.6%)	5,270 (3.5%)	2,527 (4.2%)	1,556 (4.7%)
STEMI	3,872 (0.8%)	335 (0.6%)	1,177 (0.7%)	1,338 (0.9%)	622 (1.0%)	400 (1.2%)
NSTEMI	7,913 (1.7%)	651 (1.1%)	2,414 (1.4%)	2,770 (1.8%)	1,286 (2.2%)	792 (2.4%)

Mean ± standard deviation for continuous variables and frequency and percentage for categorical variables.

### 3.2 Association between different leisure-related sedentary times and MI, STEMI, and NSTEMI incidence

**Figure 2:**
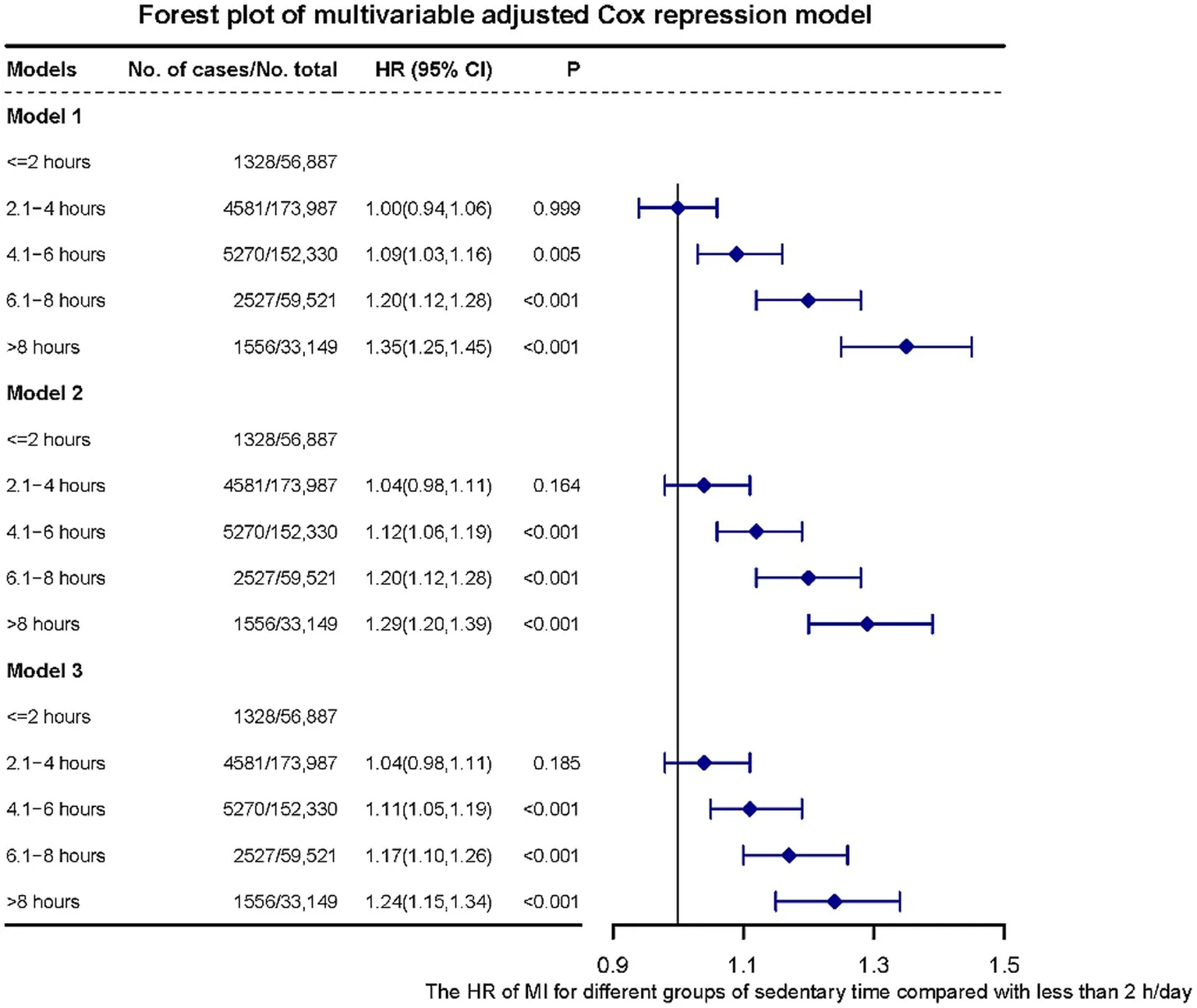
Association between different leisure-related sedentary time and MI incidence for multivariable adjusted models.

The associations of different leisure-related sedentary times with MI risk are shown in Table 2 and the graphical abstract. In the minimally adjusted model, compared with participants with sitting time≤2 h, participants with longer sitting time tend to have higher MI risk. Individuals whose sitting time is between 4.1 and 6.0 h have the lowest risk for MI incidence [HR: 1.09 (1.03, 1.16)], and those who have sitting time >8 h have the highest risk for MI [HR: 1.35 (1.25, 1.45)] (Table 2 and Figure 2). After additionally adjusting for lifestyle factors and the Townsend deprivation index, employment status, and household income, the associations between different sitting times and MI risk remained significant (Figure 2). In the fully adjusted model (Model 3), we observed a consistent association between different sitting time and MI risk (Figure 2). However, in all three models, no association was detected between sitting hours between 2.1 and 4 h and MI incidence. For STEMI, this association remained between different groups of leisure-related sedentary time and STEMI risk after adjustment for multivariable factors (Supplementary Table S1); however, those with leisure-related sedentary time between 4.1 and 6.0 h did not show increased risk for STEMI incidence. For NSTEMI incidence, we observed a similar association between different groups of leisure-related sedentary time and NSTEMI in the minimally adjusted model; however, individuals with leisure-related sedentary time between 4.1 and 6.0 h demonstrated increased NSTEMI risk in models 2 and 3 (Supplementary Table S2).

**Table 2:** Association between different leisure-related sedentary time and MI incidence.

Sedentary time	No. of cases/No. total	Model 1		Model 2		Model 3	
		HR (95% CI)	*P*	HR (95% CI)	*P*	HR (95% CI)	*P*
≤2 h	1,328 (2.3%)	Reference		Reference		Reference	
2.1–4 h	4,581 (2.6%)	1.00 (0.94,1.06)	0.999	1.04 (0.98,1.11)	0.164	1.04 (0.98,1.11)	0.185
4.1–6 h	5,270 (3.5%)	1.09 (1.03,1.16)	0.005	1.12 (1.06,1.19)	<0.001	1.11 (1.05,1.19)	<0.001
6.1–8 h	2,527 (4.2%)	1.20 (1.12,1.28)	<0.001	1.20 (1.12,1.28)	<0.001	1.17 (1.10,1.26)	<0.001
>8 h	1,556 (4.7%)	1.35 (1.25,1.45)	<0.001	1.29 (1.20,1.39)	<0.001	1.24 (1.15,1.34)	<0.001

Results of the mutually adjusted analysis are shown in Table 3. Compared with the reference category, all three alternative behaviors—sleep, LPA, and MVPA—showed inverse associations with MI risk when entered simultaneously with leisure-related sedentary time in a single Cox model. Coefficient contrasts indicated that a 60-min higher duration of sleep, LPA, or MVPA—holding sedentary time and the other two behaviors constant—was associated with HRs of 0.967 (95% CI: 0.954–0.980) for sleep, 0.987 (95% CI: 0.964–1.011) for LPA, and 0.974 (95% CI: 0.953–0.995) for MVPA. Consistent patterns were observed for 30-min and 15-min contrasts: HRs were 0.984 (95% CI: 0.977–0.990) and 0.992 (95% CI: 0.989–0.995) for sleep, 0.994 (95% CI: 0.981–1.008) and 0.998 (95% CI: 0.990–1.005) for LPA, and 0.987 (95% CI: 0.975–0.999) and 0.993 (95% CI: 0.987–1.000) for MVPA, respectively. A dose–response gradient was evident across all three magnitudes for sleep and MVPA; for LPA, point estimates were below 1.00 but 95% CIs included the null at all three time contrasts.

**Table 3:** Mutually adjusted associations of physical activity, sleep, and leisure-related sedentary behavior with incident MI.

Direction	Shift_min	HR	Lower_CI	Upper_CI
Sed → sleep*	15	0.992	0.989	0.995
Sed → sleep	30	0.984	0.977	0.990
Sed → sleep	60	0.967	0.954	0.980
Sed → LPA*	15	0.998	0.990	1.005
Sed → LPA	30	0.995	0.981	1.008
Sed → LPA	60	0.987	0.964	1.011
Sed → MVPA*	15	0.993	0.987	1.000
Sed → MVPA	30	0.987	0.975	0.999
Sed → MVPA	60	0.974	0.953	0.995

*Sed→X = replacing leisure-related sedentary time with X (sleep, LPA, MVPA); shift_min = minutes substituted.

### 3.3 Sensitivity analysis

**Figure 3:**
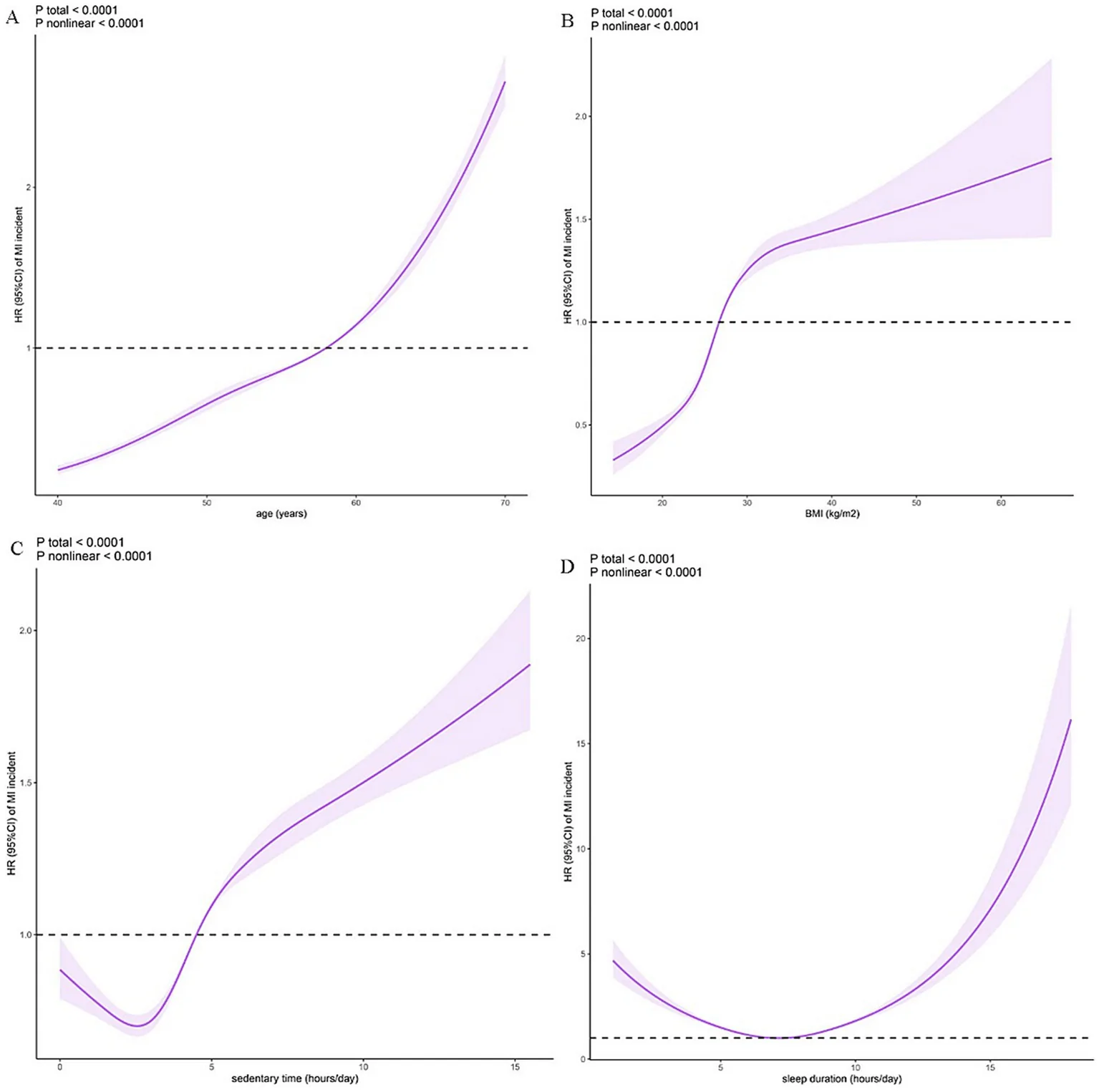
Independent associations between continuous variables and incident MI using a restricted cubic spline regression model.

To assess the functional form of key continuous variables, we used restricted cubic splines (RCS) with four knots in fully adjusted models. Leisure-related sedentary time​ exhibited a non-linear J-shaped association with MI risk (p for non-linearity < 0.0001; Figure 3C), with an inflection point at approximately 4.5 h/day, beyond which MI risk increased progressively. Sleep duration​ showed a U-shaped association with MI incidence (p for non-linearity < 0.0001; Figure 3D), with the lowest risk observed at approximately 7–7.5 h/day and elevated risks at both shorter and longer durations. Age and BMI also demonstrated significant non-linear associations with MI risk (both p for non-linearity < 0.0001; Figures 3A,B), with inflection points at approximately 58 years and 27 kg/m^2^, respectively. Similar non-linear patterns were observed for these variables in relation to STEMI and NSTEMI incidence (Supplementary Figures S2–S6).

Among participants with baseline sleep duration <7 h/day, higher sleep duration was most strongly associated with lower MI risk: 60-min, 30-min, and 15-min contrasts yielded HRs of 0.968 (95% CI: 0.956–0.981), 0.984 (95% CI: 0.978–0.990, Supplementary Table S3), and 0.992 (95% CI: 0.989–0.995), respectively. For MVPA, the corresponding HRs were 0.977 (95% CI: 0.958–0.997), 0.988 (95% CI: 0.977–0.999), and 0.994 (95% CI: 0.988–1.000). LPA point estimates were below 1.00 but did not reach statistical significance. In the 7–9 h/day group, the inverse association for sleep was attenuated: 60-min, 30-min, and 15-min contrasts produced HRs of 0.966 (95% CI: 0.953–0.979), 0.983 (95% CI: 0.977–0.989), and 0.992 (95% CI: 0.988–0.995), respectively. MVPA associations remained statistically significant (60-min HR = 0.973, 95% CI: 0.951–0.996), while LPA confidence intervals included the null. Among participants with baseline sleep duration ≥9 h/day, higher sleep duration was not associated with reduced MI risk: 60-min, 30-min, and 15-min contrasts yielded HRs of 0.965 (95% CI: 0.952–0.978), 0.983 (95% CI: 0.976–0.989), and 0.991 (95% CI: 0.988–0.995), respectively, with all confidence intervals overlapping 1.00. MVPA associations remained directionally inverse but were less precise.

In mutually adjusted models, higher durations of physical activity and sleep were independently associated with lower risks of both STEMI and NSTEMI. For STEMI, compared with shorter durations, every additional hour of sleep was associated with a 7% lower risk (HR = 0.93, 95% CI: 0.90–0.96, *p* < 0.001), and higher VPA was associated with an 8% lower risk (HR = 0.92, 95% CI: 0.85–0.99, *p* = 0.03). Associations for LPA and MPA were null (HR ≈ 0.99, *p* > 0.05). For NSTEMI, the inverse association with sleep duration was stronger, with a 5% risk reduction per additional hour (HR = 0.95, 95% CI: 0.93–0.97, *p* < 0.001). Both MPA and VPA showed significant inverse associations (HRs ≈ 0.97, *p* < 0.05), while LPA showed a modest inverse association (HR = 0.96, *p* = 0.002, Supplementary Table S4). After accounting for the competing risk of death, we observed consistent increased risks for MI across groups with sedentary time 4.1–6.0 h/day, 6.1–8.0 h/day, and >8 h/day, with HRs of 1.123 (95% CI: 1.052–1.199), 1.164 (95% CI: 1.083–1.253), and 1.232 (95% CI: 1.136–1.337), respectively (Supplementary Table S5). For STEMI and NSTEMI incidence, we observed similar patterns of association to the primary analysis (Supplementary Table S5).

## 4 Discussion

In this study, we examined the associations between leisure-related sedentary time and the incidence of myocardial infarction (MI) and its subtypes (STEMI and NSTEMI) using multivariable-adjusted and mutually adjusted Cox regression models. We found that individuals reporting more than 4 h of leisure-related sedentary time per day had an elevated risk of MI, STEMI, and NSTEMI compared with those reporting less than 2 h per day. In mutually adjusted models that simultaneously included sleep, light physical activity (LPA), and moderate-to-vigorous physical activity (MVPA), longer sleep, LPA, and MVPA were each inversely associated with MI risk, independent of sedentary time and one another. Conversely, higher sedentary time was associated with increased risk when other behaviors were held constant.

We identified a non-linear J-shaped association between leisure-related sedentary time and all three outcomes, with an inflection point at approximately 4.5 h per day, beyond which risk increased progressively. This pattern is consistent with previous investigations reporting J-shaped or threshold associations between sedentary behavior and multiple chronic conditions, including dementia (19), depression (20), irritable bowel syndrome (24), cardiovascular disease (25), and coronary heart disease (22). The consistency across diverse outcomes suggests that prolonged sedentary time may confer generalized cardiometabolic and neurological harm (26), rather than being specific to any single disease pathway.

For sleep duration, we observed a U-shaped association with MI, STEMI, and NSTEMI incidence, with the lowest risk at approximately 7–7.5 h per day and elevated risks at both shorter and longer durations. This mirrors findings from a prospective study of dementia, in which both short and long sleep durations were associated with higher hazard ratios compared with the reference group (7 h/day) (19). Our results reinforce the importance of maintaining an appropriate sleep duration as part of a healthy daily routine.

In mutually adjusted analyses, a 1-h increase in sleep duration, LPA, or MVPA—holding other measured behaviors constant—was associated with lower MI risk. The inverse associations were most pronounced for sleep and MVPA, while LPA yielded point estimates below 1.00 but with confidence intervals that included the null. These findings align with previous cohort studies that jointly modeled multiple activity domains and reported beneficial associations for higher physical activity and adequate sleep across a range of chronic diseases, including type 2 diabetes, depression, chronic liver disease, diverticular disease, and sleep disorders (18).

Several previous studies have used varying reference groups and categorization schemes for sedentary time. For example, Cao et al. (18) and Gao et al. (24) categorized sedentary time into four groups (≤2, 2.1–4, 4.1–6, and >6 h/day) to assess associations with 45 adverse health conditions and irritable bowel syndrome, respectively, while Stamatakis et al. used 0–4, 4–6, 6–8, and >8 h/day to examine all-cause and cardiovascular mortality. In the present study, we adopted a shorter reference group (<2 h/day) and classified leisure-related sedentary time into five categories (≤2, 2.1–4, 4.1–6, 6.1–8, and >8 h/day) to capture risk gradients across the full spectrum of exposure. For MI and STEMI, elevated risks emerged at >4 h/day; for NSTEMI, a 22% increase in risk was observed even at 2.1–4 h/day in fully adjusted models, and this association remained robust after accounting for the competing risk of death. These findings suggest that the threshold at which sedentary time becomes harmful may differ by MI subtype, a nuance that warrants consideration in future clinical and public health guidelines.

A key strength of this study is the simultaneous modeling of multiple daily behaviors within a single mutually adjusted framework. Rather than examining each behavior in isolation, this approach acknowledges that daily time use comprises interrelated components. However, several limitations must be acknowledged. First, the observational design precludes causal inference. Second, behavioral exposures were self-reported only at baseline; changes over time during follow-up were not captured. Third, the cohort consisted predominantly of individuals of European ancestry, limiting generalizability to other populations. Fourth, sedentary time was operationalized as the sum of television viewing, computer use, and driving—capturing only leisure-related sedentary behavior—while occupational and other sedentary domains were not assessed due to data availability. Fifth, physical activity was derived from self-reported questionnaire items; walking was classified as light-intensity activity following standard conventions, though its actual intensity may vary. Sixth, although we adjusted for cardiometabolic medication use and body mass index as proxies for dyslipidemia and chronic kidney disease, direct clinical measures of these conditions were unavailable, and residual confounding from unmeasured factors (e.g., family history and diet quality) cannot be entirely ruled out. Seventh, the measured behaviors do not constitute a complete 24-h compositional dataset; unmeasured residual time was not explicitly modeled; therefore, the present analysis should not be interpreted as formal isotemporal substitution.

In conclusion, prolonged leisure-related sedentary time is associated with increased risks of MI, STEMI, and NSTEMI. Independent of other daily behaviors, longer sleep and physical activity durations are inversely associated with MI incidence. Future public health guidelines should consider subtype-specific thresholds and promote multidimensional behavioral strategies—encompassing adequate sleep, reduced leisure sedentary time, and increased physical activity—for cardiovascular disease prevention.

## Data Availability

The data used in this study is publicly available in an open access repository in the UK Biobank Resource under application number 772449. These data cannot be shared with other investigators.
