# Supplementary material for: Leisure-related sedentary time and physical activity with myocardial infarction incidence: a prospective cohort study
Source: Front Nutr. 2026 Sep 9;13:1902990. doi: 10.3389/fnut.2026.1902990 (PMC13597396; doi:10.3389/fnut.2026.1902990)
Supplement: Supplementary file 1 [file Data_Sheet_1.DOCX]

Supplementary Material

# 1 Supplementary Figures and Tables

## 1.1 Supplementary Figures

**Figure S1**. Directed Acyclic Graph (DAG) illustrating the prespecified causal framework for 24-hour behavior and incident myocardial infarction.

**Figure S2.** The association between different leisure-related sedentary behaviors and MI incidence using restricted cubic spline fitted cox regression model.

**Figure S3 The independent associations between continuous variables and incident STEMI using a restricted cubic spline regression model.**

**Figure S4 The independent associations between continuous variables and incident NSTEMI using a restricted cubic spline regression model.**

**Figure S5 The association between different leisure-related sedentary behaviors and STEMI incidence using restricted cubic spline fitted cox regression model.**

**Figure S6 The association between different leisure-related sedentary behaviors and NSTEMI incidence using restricted cubic spline fitted cox regression model.**

## 1.3 Supplementary Tables

**Table S1 Association between different leisure-related sedentary time with STEMI incidence.**

| Sedentary time | No. of cases/No. total | Model 1 | | Model 2 | | Model 3 | |
| --- | --- | --- | --- | --- | --- | --- | --- |
|  |  | **HR (95% CI)** | ***P*** | **HR (95% CI)** | ***P*** | **HR (95% CI)** | ***P*** |
| ≤2 hours | 335/56,887 | Reference |  | Reference |  | Reference |  |
| 2.1-4 hours | 1177/173,987 | 1.04 (0.92, 1.17) | 0.562 | 1.07 (0.95, 1.21) | 0.268 | 1.07 (0.95, 1.21) | 0.266 |
| 4.1-6 hours | 1338/152,330 | 1.15 (1.02, 1.30) | 0.022 | 1.17 (1.03, 1.32) | 0.014 | 1.16 (1.03, 1.32) | 0.014 |
| 6.1-8 hours | 622/59,521 | 1.24 (1.08, 1.42) | 0.002 | 1.22 (1.07, 1.40) | 0.004 | 1.22 (1.06, 1.39) | 0.005 |
| >8 hours | 400/33,149 | 1.41 (1.22, 1.63) | <0.001 | 1.34 (1.16, 1.56) | <0.001 | 1.33 (1.14, 1.54) | <0.001 |

**Table S2 Association between different leisure-related sedentary time with NSTEMI incidence.**

| Sedentary time | No. of cases/No. total | Model 1 | | Model 2 | | Model 3 | |
| --- | --- | --- | --- | --- | --- | --- | --- |
|  |  | **HR (95% CI)** | ***P*** | **HR (95% CI)** | ***P*** | **HR (95% CI)** | ***P*** |
| ≤2 hours | 651/56,887 | Reference |  | Reference |  | Reference |  |
| 2.1-4 hours | 2414/173,987 | 1.07 (0.98, 1.17) | 0.11 | 1.11 (1.02, 1.21) | 0.02 | 1.11 (1.02, 1.21) | 0.019 |
| 4.1-6 hours | 2770/152,330 | 1.17 (1.07, 1.27) | <0.001 | 1.19 (1.09, 1.29) | <0.001 | 1.18 (1.08, 1.29) | <0.001 |
| 6.1-8 hours | 1286/59,521 | 1.24 (1.13, 1.36) | <0.001 | 1.22 (1.11, 1.34) | <0.001 | 1.20 (1.09, 1.32) | <0.001 |
| >8 hours | 792/33,149 | 1.39 (1.25, 1.54) | <0.001 | 1.31 (1.18, 1.46) | <0.001 | 1.27 (1.14, 1.41) | <0.001 |

**Table S3 Mutually adjusted inter-behavior associations with incident MI by baseline sleep duration**

| shift_min | HR | lower_CI | upper_CI | sleep_group | direction |  |
| --- | --- | --- | --- | --- | --- | --- |
| 15 | 0.992 | 0.989 | 0.995 | <7h | SED_to_Sleep | |
| 30 | 0.984 | 0.978 | 0.990 | <7h | SED_to_Sleep | |
| 60 | 0.968 | 0.956 | 0.981 | <7h | SED_to_Sleep | |
| 15 | 0.998 | 0.992 | 1.005 | <7h | SED_to_LPA | |
| 30 | 0.996 | 0.984 | 1.008 | <7h | SED_to_LPA | |
| 60 | 0.989 | 0.969 | 1.011 | <7h | SED_to_LPA | |
| 15 | 0.994 | 0.988 | 1.000 | <7h | SED_to_MVPA | |
| 30 | 0.988 | 0.977 | 1.000 | <7h | SED_to_MVPA | |
| 60 | 0.977 | 0.958 | 0.997 | <7h | SED_to_MVPA | |
| 15 | 0.992 | 0.988 | 0.995 | 7-9h | SED_to_Sleep | |
| 30 | 0.983 | 0.977 | 0.990 | 7-9h | SED_to_Sleep | |
| 60 | 0.966 | 0.953 | 0.979 | 7-9h | SED_to_Sleep | |
| 15 | 0.998 | 0.990 | 1.005 | 7-9h | SED_to_LPA | |
| 30 | 0.994 | 0.981 | 1.008 | 7-9h | SED_to_LPA | |
| 60 | 0.987 | 0.963 | 1.011 | 7-9h | SED_to_LPA | |
| 15 | 0.993 | 0.986 | 1.000 | 7-9h | SED_to_MVPA | |
| 30 | 0.986 | 0.974 | 0.999 | 7-9h | SED_to_MVPA | |
| 60 | 0.973 | 0.951 | 0.995 | 7-9h | SED_to_MVPA | |
| 15 | 0.991 | 0.988 | 0.995 | >=9h | SED_to_Sleep | |
| 30 | 0.983 | 0.976 | 0.989 | >=9h | SED_to_Sleep | |
| 60 | 0.965 | 0.952 | 0.978 | >=9h | SED_to_Sleep | |
| 15 | 0.998 | 0.989 | 1.007 | >=9h | SED_to_LPA | |
| 30 | 0.995 | 0.978 | 1.011 | >=9h | SED_to_LPA | |
| 60 | 0.986 | 0.958 | 1.014 | >=9h | SED_to_LPA | |
| 15 | 0.992 | 0.984 | 1.000 | >=9h | SED_to_MVPA | |
| 30 | 0.985 | 0.970 | 1.000 | >=9h | SED_to_MVPA | |
| 60 | 0.970 | 0.945 | 0.995 | >=9h | SED_to_MVPA | |

** denotes *P*<0.01

**Table S4 Associations of PA and sleep with incident STEMI/NSTEMI by replacing SB in the mutually adjusted model in the whole population**

| Outcomes | Activity | HR (95% CI) | *P* |
| --- | --- | --- | --- |
| STEMI | LPA | 0.99 (0.96, 1.02) | 0.51 |
|  | MPA | 0.98 (0.95, 1.02) | 0.34 |
|  | VPA | 0.92 (0.85, 0.99) | 0.03 |
|  | Sleep duration | 0.93 (0.90, 0.96) | <0.001 |
| NSTEMI | LPA | 0.96 (0.94, 0.99) | 0.002 |
|  | MPA | 0.97 (0.95, 0.99) | 0.014 |
|  | VPA | 0.97 (0.95, 0.99) | 0.012 |
|  | Sleep duration | 0.95 (0.93, 0.97) | <0.001 |

**Table S5 Association between different leisure-related sedentary time with different outcomes using competing risk model.**

| Outcomes | Sedentary time | Model 1 | | Model 2 | | Model 3 | |
| --- | --- | --- | --- | --- | --- | --- | --- |
|  |  | **HR (95% CI)** | ***P*** | **HR (95% CI)** | ***P*** | **HR (95% CI)** | ***P*** |
| MI | ≤2 hours | Reference |  | Reference |  | Reference |  |
|  | 2.1-4 hours | 1.06 (0.993, 1.131) | 0.082 | 1.056 (0.989, 1.128) | 0.100 | 1.055 (0.988, 1.127) | 0.110 |
|  | 4.1-6 hours | 1.203 (1.127, 1.283) | 2.30E-08 | 1.127 (1.056, 1.204) | 3.30E-04 | 1.123 (1.052, 1.199) | 5.10E-04 |
|  | 6.1-8 hours | 1.353 (1.259, 1.453) | 2.20E-16 | 1.182 (1.099, 1.272) | 6.50E-06 | 1.164 (1.083, 1.253) | 4.20E-05 |
|  | >8 hours | 1.558 (1.439, 1.687) | <0.0001 | 1.271 (1.172, 1.378) | 7.10E-09 | 1.232 (1.136, 1.337) | 4.90E-07 |
| STEMI | ≤2 hours | Reference |  | Reference |  | Reference |  |
|  | 2.1-4 hours | 1.044 (0.919, 1.19) | 0.510 | 1.055(0.928, 1.2) | 0.410 | 1.057 (0.929, 1.202) | 0.400 |
|  | 4.1-6 hours | 1.181 (1.04, 1.34) | 0.010 | 1.156 (1.017, 1.315) | 0.027 | 1.158 (1.018, 1.317) | 0.026 |
|  | 6.1-8 hours | 1.272 (1.104, 1.47) | 8.80E-04 | 1.2 (1.039, 1.386) | 0.013 | 1.199 (1.038, 1.384) | 0.014 |
|  | >8 hours | 1.471 (1.26, 1.72) | 1.00E-06 | 1.333 (1.138, 1.563) | 3.80E-04 | 1.324 (1.13, 1.552) | 5.30E-04 |
| NSTEMI | ≤2 hours | Reference |  | Reference |  | Reference |  |
|  | 2.1-4 hours | 1.143 (1.042, 1.254) | 4.60E-03 | 1.129 (1.029, 1.239) | 0.011 | 1.129 (1.029, 1.239) | 0.011 |
|  | 4.1-6 hours | 1.296 (1.182, 1.421) | 3.40E-08 | 1.196 (1.09, 1.313) | 1.60E-04 | 1.193 (1.087, 1.31) | 2.00E-04 |
|  | 6.1-8 hours | 1.419 (1.281, 1.571) | 1.70E-11 | 1.217 (1.098, 1.35) | 1.90E-04 | 1.202 (1.084, 1.333) | 4.70E-04 |
|  | >8 hours | 1.573 (1.405, 1.762) | 4.90E-15 | 1.256 (1.119, 1.411) | 1.10E-04 | 1.221 (1.087, 1.371) | 7.60E-04 |
